# Locomotor Ataxia

**Published:** 1867-11

**Authors:** 


### LOCOMOTOR ATAXIA.

(Translated, from the German of Althaus in the Deutsches Klink, 1866, for the *Quarterly Journal of Psychological Medicine and Medical Jurisprudence.*)

We may adopt, with Duchenne, three stages of this disease. The first lasts from four to five years, and is characterized by troubles of the cranial nerves, by peculiar pains, and a decrease of the generative power. The second stage lasts ten years or more, and is signalized by the appearance of ataxia and decreasing sensibility. To these symptoms is to be added paralysis in the third stage, and death ensues by exhaustion or some intercurrent disease. The affection is from the beginning either chronic or subacute. The cranial nerves are first attacked, *i.e.*, the optic, the motores oculorum, trochlearis, the abducentes, (amblyopia, diplopia, strabismus, ptosis). The ophthalmoscope ascertains the symptoms of congestion (expansion of the capillary vessels and violet coloring of the membrane) and afterwards (in amaurosis) an atrophy of the retnai. As to its prognosis, it is to be remarked that strabismus or diplopia may disappear even without treatment, while ptosis or amblyopia will not. The pains of such patients are described as jerking, suddenly appearing and disappearing. They are generally located in the lower extremities, and are paroxysmal. If the pain occurs in the bladder, a vesicular catarrh will soon supervene. These pains diminish in spring, but increase after fatiguing walks, and especially after venereal and alcoholic excesses. An important, but not constant symptom, is spermatorrhœa, first with, and afterwards without erections; exceptionally, priapismus and satyriasis will occur. There is sometimes, in the second stage, besides strangury, incontinence of urine and inability to retain the feces, and symptoms of ataxia, appear on a sudden or by degrees. The still vigorous muscles react no longer normally upon the emotional impulse, the equilibrium is lost, complicated motions can no longer be effected. There are two coördinate kinds of muscular activity, *viz.:* the harmony between the antagonists, and the instinctive, or emotional muscular actions. In the latter the antagonists do not remain inactive, but every intended motion results from a twofold nervous action, so that one group of muscles produces a motion, while another moderates it. In progressive ataxia, the capacity for controlling the muscular actions is lost first, although every single muscle may still be contracted, and though even certain associated motions may succeed. Separate muscular contractions, however, do not occur in a normal condition; most of the muscular actions require a great number of simultaneous muscular contractions to which we accustomed ourselves from childhood, and every child suffers, as it were, by ataxia, without being aware of it. Now these associated muscular actions take place in adults instinctively without any special emotional impulse, and if this capacity is gone, we have a case of ataxia before us. It begins mostly in the lower extremities, the gait of the patient becomes faltering (as with many inebriates) and this infirmity is constantly on the increase; if you cause the patient to stand on his feet joined closely together, he begins to sway to and fro, particularly when his eyes are closed. Still this swaying does not in every case indicate ataxia, since it also occurs in different diseases of the brain, with feeble, very anemic persons and infirm convalescents. These symptoms increase by walking, especially when the patient wishes to turn around, in which case the feet are tossed to and fro without purpose, and become useless. Irregular muscular contractions will also take place.

The patient is, while lying down, able to stretch and bend his feet, but he does so suddenly by fits and starts, and can no longer calculate the strength required. The differences from paralysis are obvious: sometimes there is weakness of motion connected with a sensible use of it, sometimes there is sufficient, or even abundant strength, without any power of using it to some definite purpose. These symptoms are far less marked in the upper extremities, the muscles of which may be tested by causing the patient to touch his nose or to make the sign of a cross while he keeps his eyes shut. Sensibility appears disturbed, the feet seem benumbed and heavy, with or without loss of the cutaneous sensibility, this loss manifesting itself in the soles and passing over to the chest, stomach, and the ulnar side of the upper extremities. In ataxia the soles and legs are almost constantly benumbed; this not being so, renders a diagnosis of ataxia doubtful. The decrease of this abnormal feeling indicates improvement. Anæsthesia occurs frequently, but only at a late period and not constantly, but the sensibility of touch never becomes extinct (analgesia, anodynia). The sense of touch is frequently everywhere diminished, and the patient needs often several minutes before he perceives the impression of touch; the reflex movements are, therefore, defective or entirely wanting. The influence of temperature is seldom impaired, but the patient may become unconscious of it, for it is known that patients, supposing they were going to take a tepid bath, have been scalded. The ability of distinguishing weight, too, is diminished, for such as suffer by ataxia cannot, like healthy persons, discriminate between a weight of 29lbs. in one hand and 30lbs. in the other. As this ability may, in the soles of the feet, be diminished, so that the gait becomes faltering, the diminution or absence of this ability has likewise been considered the cause of the faltering gait of persons attacked by ataxia. All the symptoms mentioned above, increase in the last stage of this disease, while the muscular power decreases; the muscles become atrophied, convulsions take place, amamosis, palsy of the bladder, or some intercurrent disease put an end to the sufferings of the patient. It is a singular fact that the disposition of mind of such patients is quite cheerful, notwithstanding all their sufferings.

Regarding the *causes* of this disease we observe, that the greater part of the patients were from thirty to fifty years old, and that this disease affects more men than women. Hardship, cold, and dampness are rightly considered to produce it. The suffering is almost constantly aggravated in autumn and winter:

if there is an improvement in this period, it may properly be attributed to the treatment of the disease. It is not yet ascertained, whether venereal excesses are connected with the origin of this disease, they are at any rate not its principal cause, as was formerly thought to be the case. Over exertion and nervous diseases of the parents are certainly additional causes.

The *diagnosis*, doubtful in the first stage, becomes very easy in the second, even if all symptoms other than ataxia of the muscles are wanting; in the third stage ataxia does no longer exist by itself, as the degeneration of the spinal marrow extends already to the spinal arteries. Ataxia *might be mistaken* for: 1. *myelitis*, in which, however, palsies may occur, but not ataxia; there is no sympathetic affection of the cranial nerves, but pain in the back, spasms and muscular atrophy occur. 2. *Diseases of the cerebellum*, in which a condition similar to ataxia may appear, although the inclination to running forward or backward is more frequently noticed. Important distinctive symptoms are the fixed pain in the occiput, vomiting, a more indolent affection of the cranial nerves, convulsions and the epileptoid symptoms, all of which are absent in ataxia. 3. *Softening of the brain*, in which, however, hemiplegia, loss of memory and mind take place. 4. *Chronic, poisoning by alcohol, lead and mercury* as well as syphilitic affections of the brain, in which diseases we must use anamnestic medicines and apply a treatment *ex juvantibus et nocentibus.* 5. *Palsy of the muscular sense*, a symptom common to different affections; but here the patient is completely palsied, as soon as the eye (representing the muscular sense) does not act any longer; as soon as the patient shuts his eyes or is in the dark, he is palsied; the muscular power is retained, as in ataxia, but the patient can effect motion only in daylight and with open eyes.

*Prognosis. Romberg's* despairing warning against any therapeutics as merely aggravating the disease, has been repudiated by experience. Even in advanced cases the sufferings of the patients may still be considerably alleviated. Intercurrent diseases impair the prognosis. Two facts encourage the physician to therapeutic interference: first, that the symptoms affecting the cranial nerves (excepting the optic) disappear in time, so that their exists a functional stage of the disease, before the physical changes in the spinal marrow occur, in which stage medicines may accomplish much; second, that Charcot and Vulpian found, in a case of ataxia, some nerve fibres in the spinal marrow which were evidently in a stage of regeneration.

*Treatment.* If the patient is feeble, declining anemic, prescribe, besides nutritious food, iron, quinine, cod-liver oil, and milk, which latter Hippocrates has recommended for erotic tabes. If great exertions have preceded, rest is imperatively required. Counter-irritants applied to the back must be wholly avoided, but the constant electric fluid may be passed along the vertebral column. Iodine should not be employed, but iodide of potassium and iron may be used, although they may not cure the disease. Baths may be recommended, but ataxic persons should not travel a great distance for them, but rather drink mineral water and take sulphuretted baths at home. Faradization is of no use, galvinization avails against some symptoms without curing the disease. The most reliable of all curative agents is still the arg. nitricum given at the rate of $\frac{1}{10}-\frac{1}{2}$ gr. two or three times a day, which may be administered in connection with phosphate of lime (10–20 gr. pro die). This medicine may in such a dose be given without danger, especially if you discontinue it for a fortnight after every four weeks and give a purgative instead. I pay close attention to the condition of the gums. We must, in general, take care not to treat this disease according to one rule, but sharply individualize, as in all other diseases.

